# An Attomolar-Level Optical Device for Monitoring Receptor–Analyte Interactions Without Functionalization Steps: A Case Study of Cytokine Detection

**DOI:** 10.3390/s25030930

**Published:** 2025-02-04

**Authors:** Nunzio Cennamo, Francesco Arcadio, Chiara Marzano, Rosalba Pitruzzella, Mimimorena Seggio, Maria Pesavento, Stefano Toldo, Antonio Abbate, Luigi Zeni

**Affiliations:** 1 Department of Engineering, University of Campania Luigi Vanvitelli, Via Roma 29, 81031 Aversa, Italy; francesco.arcadio@unicampania.it (F.A.); chiara.marzano@unicampania.it (C.M.); rosalba.pitruzzella@unicampania.it (R.P.); mimiseggio@gmail.com (M.S.); luigi.zeni@unicampania.it (L.Z.); 2 Optosensing Srl, Via Carlo de Marco 69F, 80137 Naples, Italy; maria.pesavento@optosensing.it; 3 Berne Cardiovascular Research Center and Division of Cardiovascular Medicine, University of Virginia, Charlottesville, VA 22908, USA; toldostefano@gmail.com (S.T.); antonio.abbate@virginia.edu (A.A.)

**Keywords:** plasmonic sensors, plastic optical fibers (POFs), optical biosensors, surface plasmon resonance (SPR), receptor–analyte interactions, cytokine monitoring, attomolar-level detection

## Abstract

A plastic optical fiber (POF)-based device for biosensing strategies has been developed to monitor several protein–protein interactions at ultra-low concentrations without functionalization processes, exploiting plasmonic phenomena. In this work, novel tests were applied to different kinds of analyte–receptor interactions, such as interleukins, where the bioreceptor’s (protein antibody) molecular weight is roughly ten times that of the analyte (protein interleukin), while intracellular bioreceptors and small molecules at low molecular weight interactions have already been demonstrated via the same point-of-care test (POCT). The POCT was implemented by a white light source and a spectrometer connected via two POF-based chips connected in series: an innovative microcuvette chip and a D-shaped POF surface plasmon resonance (SPR) probe. In particular, the POF microcuvette chip was achieved by drilling three micro holes in the core of a modified POF. Instead of performing a functionalization step, the micro holes were filled with a specific receptor solution for the analyte (one microliter at the femtomolar level), which selectively captured the target (e.g., cytokine) when the samples were dropped over the filled micro holes (twenty microliters at the attomolar level). Three interleukins, IL-1β, IL-17A, and IL-18, were detected in the attomolar concentrations range by monitoring the resonance wavelength shift over time due to the cytokine/antibody (protein–protein) interaction. The POF-based device was proven to be effective for detecting several interleukins at the attomolar level in a few minutes and without functionalization processes.

## 1. Introduction

Cytokines are a broad and loose category of proteins involved in cell signaling that play a central role in coordinating and modulating immune, inflammatory, and reparative responses in the human body [1]. In particular, these macromolecules are not only involved in the physiological defense against infections and pathogens but also play a significant role in several patho-physiological processes, including autoimmune diseases, chronic inflammatory conditions, cardiovascular diseases, and cancers [2]. Their presence in the biological fluids acts as a biomarker for various diseases, and the concentration monitoring of this family of proteins in the blood and biological fluids is crucial during the diagnosis, the tracking of the disease progression, and therapeutic efficacy assessment [3]. The cytokine superfamily includes interleukins, which are a class of mediators secreted mainly by leukocytes as well as some other body cells.

The interleukins can be classified into different families based on their receptor or general function [4]. In particular, the IL-1 family is primarily associated with innate immunity but also plays a role in acquired immunity [5]. IL-1β and IL-18 belong to this class. IL-1β is a pro-inflammatory cytokine primarily produced by activated macrophages, monocytes, and dendritic cells [5]. It mediates acute inflammatory responses and promotes the release of other inflammatory mediators, amplifying the inflammatory cascade. IL-1β levels increase in several inflammatory-based diseases, such as rheumatoid arthritis [6], allergic rhinitis [7], and sepsis [8], and several types of cancer, like breast and lung tumors [9].

IL-18, originally known as interferon-gamma (IFN-γ) inducing factor, has a unique distribution and constitutive production. Indeed, many cell types, both hematopoietic and non-hematopoietic, such as Kupffer cells (liver-resident macrophages), intestinal epithelial cells, keratinocytes, and endothelial cells, have the potential to produce IL-18. The latter intervenes in host defense against various infectious microorganisms, inducing IFN-γ, nitric oxide (NO), and reactive oxygen species (ROS) in phagocytes. In addition, IL-18 directly activates CD8^+^ T cells, which are involved in viral clearance [10].

The IL-17 family comprises six proteins (from IL-17A to IL-17F). IL-17A was the first of the family to be identified and targeted in a clinical setting. The main role of this interleukin is the regulation of the interaction between commensal microorganisms and epithelial cells at the body barriers (i.e., skin and mucosae), maintaining its integrity. Moreover, IL-17 production is involved in immune-mediated inflammatory disorders and cancer [11].

The concentration ranges of interest for these cytokines vary according to the interleukin type and physio-pathological condition. For instance, IL-1β is typically detected between 0.5 and 12 pg/mL in the plasma of healthy individuals, but the concentration levels can rise significantly under pathological conditions [12]. IL-17 and IL-18 exhibit variable baseline levels, ranging from 0.7 to 26 pg/mL [13] and 80 to 120 pg/mL [14], respectively, with increases linked to inflammatory and autoimmune conditions.

Typically, interleukin detection is performed by the enzyme-linked immunosorbent assay (ELISA), which, so far, is considered the gold standard technique in cytokine determination [15]. Nevertheless, the ELISA is expensive and time-consuming, thus making this method unsuitable to be used as point-of-care tests (POCTs). In this frame, alternative methods for cytokine measurements are based on electrochemical techniques [16,17,18], fluorescence [19] and plasmonic phenomena, such as surface plasmon resonance (SPR), localized surface plasmon resonance (LSPR) and hybrid [20,21,22]. For instance, Cennamo et al. recently proposed a POCT based on a conventional SPR sensor in plastic optical fibers (POFs) to detect IL-1β in the picomolar concentration range via a specific bioreceptor [22]. Interesting results, in terms of ultra-wide and ultra-low detection ranges, were achieved for IL-6 detection via two plasmonic probes combined with the same specific bioreceptor layer [21]. However, the above-mentioned sensing solutions require functionalization processes of the sensitive surface [17,18,19,20,21,22].

In this work, a POF-based device is presented to monitor several interleukin–antibody interactions at ultra-low concentrations (at the attomolar level) without functionalization processes, exploiting an innovative sensing strategy, based on multimode POFs and an unconventional plasmonic scheme, presented in [23] for the detection of cortisol and estradiol. More specifically, the microcuvette-based device in [23] was employed to monitor the interaction between intracellular bioreceptors (estrogen receptor and glucocorticoid receptor) and small molecules at low molecular weights (estradiol and cortisol, respectively). Instead, in this work, for the first time, the interaction between antibodies (protein) and cytokines (protein) was monitored. The monitoring of the protein–protein interaction has never been tested before by means of the microcuvette-based device. In particular, the aim of the present work is to demonstrate that the previously proposed sensing method [23] can be safely applied to different kinds of analyte–receptor interactions, such as interleukins. In these novel device tests, the bioreceptor’s (protein antibody) molecular weight is about ten times that of the analyte (protein interleukin). In particular, the device consists of a sensitive POF microcuvette chip and an SPR-POF probe connected in series. More specifically, the POF-based microcuvette chip is achieved by drilling three micro holes in a D-shaped POF region directly in the core of a modified POF. The microcuvettes are filled with a specific receptor solution, which selectively recognizes the target analyte in the samples dropped over the filled micro holes, avoiding immobilization procedures. Any variation occurring in the micro holes due to the molecule binding processes changes the mode profile of the propagated light in the multimode POF’s core, modulating the plasmonic phenomena in the subsequent SPR-POF probe [23]. In this work, three interleukins, IL-1β, IL-18, and IL-17A, were detected in the attomolar concentration range to demonstrate the device’s capabilities. Furthermore, selectivity tests were performed to assess the specificity of the presented sensing solution.

## 2. Materials and Methods

### 2.1. Chemicals

MilliQ water and phosphate-buffered saline (PBS) were purchased from Sigma-Aldrich. Recombinant human IL-1β protein (active form, 17,000 Da) (ab259387); recombinant human IL-18 protein (ab316093); recombinant human IL-17A protein (ab282392); rabbit recombinant monoclonal anti-IL-1β antibody [EPR21086] (ab216995); rabbit recombinant monoclonal anti-lL-18 antibody [EPR19954-188] (ab243091); and rabbit polyclonal anti-IL-17A antibody (ab79056) were purchased from Abcam (Cambridge, UK).

### 2.2. Fabrication of the POF-Based Sensitive Patch with Micro Holes

The fabrication of the POF-based sensitive microcuvette chip was carried out according to the procedure reported in [23,24]. More specifically, a 1 mm POF (980 μm core and 10 μm cladding, manufactured by Edmund Optics, Barrington, NJ, USA) was embedded in a resin block and polished with two paper sheets with decreasing grits (5 μm and 1 μm) in order to obtain a D-shaped POF region with the exposed core. Then, three micro holes with a diameter equal to about 600 μm and a depth of about 500 μm were drilled into the D-shaped POF in an orthogonal orientation with respect to the direction of the propagating light. The fabrication procedure was carried out by a computer numerical control (CNC) micro-milling machine.

### 2.3. SPR-POF Probe Fabrication

The SPR-POF platform fabrication is detailed in [25]. In summary, the same kind of multimode POF previously mentioned (see Section 2.2) was glued into a resin support with a trench of suitable dimensions at the surface and then polished with two lapping papers to realize the D-shaped POF sensitive region with a length of 10 mm [25]. A photoresist buffer layer (Microposit S1813, MicroChem Corp., Westborough, MA, USA) was spin-coated on the exposed core in the D-shaped POF region to improve the optical performance and the subsequent gold film adhesion [25]. Finally, a 60 nm gold film was deposited by a sputter coater machine (Safematic CCU-010, Zizers, Switzerland), obtaining a planar plasmonic surface.

### 2.4. Experimental Setup

**Figure 1:**
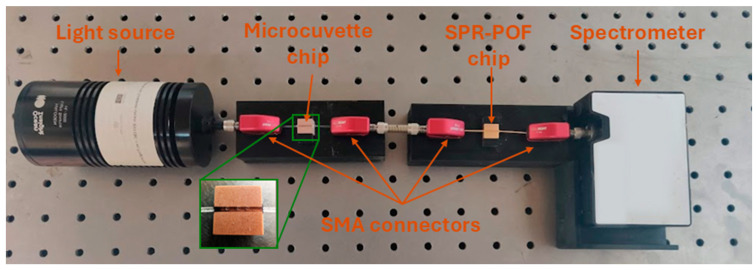
Actual image of the experimental setup. In the zoom the POF-based microcuvette chip (with three micro holes in the D-shaped POF area).

During the measurements carried out with the microcuvette-based chip, the SPR-POF platform was located between the sensing POF microcuvette chip and the spectrometer, as described in [23]. A picture of the experimental setup is reported in Figure 1. It consisted of a white light source (HL2000-LL, Ocean Insight, Orlando, FL, USA) illuminating the POF-based sensitive patch with micro holes, where the antibody–cytokine binding takes place into the microcuvette. The latter chip was used to launch the light into the SPR-POF probe with the SPR surface in contact with a fixed solution with a suitable refractive index (RI = 1.332, i.e., water) used to trigger the SPR phenomena. The light transmitted by the plasmonic platform was then collected by a spectrometer (FLAME-S-VIS-NIR-ES, Ocean Insight, Orlando, FL, USA), which was connected to a laptop to acquire and process the experimental data. The setup implemented a measuring scheme that harnessed the SPR technique in a way that is different from the classical methodology. The sensor system did not exploit the refractive index variation due to the receptor–analyte interactions at the plasmonic surface, but it exploited the characteristics of the multimode waveguides (in this case, the modified POFs) that can be used to achieve a variation of the incident angles of the propagated light under the other D-shaped POF chip with an SPR surface as a consequence of the receptor–analyte interactions in the core of the D-shaped POF with the micro holes, causing a shift in the resonance wavelength [23]. The device presented two D-shaped POF chips (microcuvette and SPR chips) connected via SMA connectors; so, starting from the primary exciting light source, there were different changes in the propagated modes, i.e., an intra-POF (from a circular POF to a D-shaped POF and from the D-shaped POF to a circular POF) considering the first chip. Then, the latter acted as the exciting source connected to the second chip via a free-space propagation region (the metal tube screwed to the SMA connectors). This means that the light impinges on the input fiber of the second chip (the plasmonic one) with a modal configuration that depends on the status of the first chip.

### 2.5. Ligand–Receptor Binding Measurement Protocols and Data Processing

**Figure 2:**
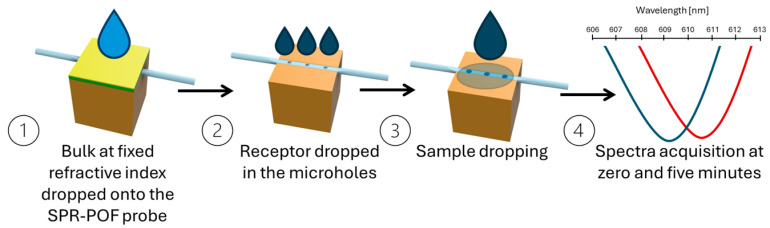
Outline of the measurement protocol to achieve the transmitted spectra during the time. The transmitted spectra are normalized on the reference spectrum to obtain the SPR spectra (step 4).

For all the interleukin–antibody interactions, the measurements were carried out according to the measurement protocol reported by Cennamo et al. [23] and outlined in Figure 2. To summarize, at first, the reference spectrum was acquired by considering air in contact with the SPR surface (in this case, the SPR condition was not satisfied) and the micro holes in the sensing patch filled with PBS (the matrix in which the binding was investigated). The SPR condition was satisfied by dropping 50 μL of the selected dielectric (water; RI = 1.332) over the plasmonic surface of the SPR-POF probe (the gold nanofilm). The SPR spectra were obtained by normalizing the acquired transmitted spectra during the time to the reference spectrum.

The stock solutions of the three different cytokines (IL-1β, IL-18, and IL-17A) were prepared at 5 µM in PBS. The solutions at different concentrations, ranging from 5 aM to 50 fM, were prepared by diluting the stock solution with PBS. Following the protocol reported in [23], for the measurement, 20 μL of the samples were placed on the planar surface of the microcuvette sensing chip, with the three micro holes previously filled with 1 μL of receptor solution diluted in PBS at a concentration of 7.3 fM. The SPR spectra were acquired during incubation at 0 (t_0_) and 5 min (t_5_). The resonance wavelength at t_0_ was assumed as the blank for calculating the resonance wavelength variation at the equilibrium condition reached at 5 min: (Δλ_c_ = Δλ_t5_= λ_t5_ − λ_t0_) [23].

In this kind of device, only the resonance wavelength variation over time is considered, while the intensity of the spectra has no specific meaning. In particular, each response (i.e., the resonance wavelength variation) is relative and calculated with respect to its blank (solution at t = 0 min) over time. In fact, the analyte concentration “*c*” is proportional to the relative variation in resonance wavelength over time (Δλ_c_ = λ_t5_ − λ_t0_). In other words, a different blank with a different intensity and/or resonance wavelength is considered for each test. After each measurement, the micro holes were emptied and washed with PBS in order to reuse the device. During the experimental procedure, the environmental temperature was maintained fixed and controlled at 25 °C.

The absolute values of the experimental data ($\left|{\Delta \lambda }_{c}\right|$) were reported as a function of the interleukin concentration (*c*) and fitted with the Langmuir model equation reported in Equation (1) [26].

(1) $$\left|{\Delta \lambda }_{c}\right|=\left|\Delta \lambda_{max}\right|\cdot \left(\frac{c}{K+c}\right)$$

where Δλ_c_ is evaluated as the difference between the wavelength resonance at t_5_ (equilibrium condition), named λ_t5_, and that at t_0_ (blank), named λ_t0_. $\left|\Delta \lambda_{max}\right|$ is the |Δλ_c_| value at the saturation condition, and K is the Langmuir constant.

The error bars were calculated as the maximum experimentally measured standard deviation, obtained by testing three similar platforms in similar conditions, and were found to be equal to about 0.2 nm. The fitting was carried out by the Langmuir model using OriginPro software (version 9.2, Origin Lab. Corp., Northampton, MA, USA).

The sensor’s analytical parameters in interleukin detection were calculated as the sensitivity at a low concentration (*S_lowc_*), the limit of detection (LOD), and the affinity constant (*K_aff_*) using Equations (2)–(4), respectively [27]. In Equation (3), the standard deviation of the experimental data $\left|{\Delta \lambda }_{c}\right|$ at the blank ($\left|{\Delta \lambda }_{0}\right|$) is indicated as σ|Δλ_0_|.

(2) $$S_{lowc}=\left|\Delta \lambda_{max}\right|/K$$

(3) $$LOD=\frac{3\times \sigma_{{|\Delta \lambda }_{0}|}}{S_{lowc}}$$

(4) $$K_{aff}=\frac{1}{K}$$

## 3. Results and Discussion

In order to evaluate the response of the sensor system at different concentrations of the cytokines (IL-1β, IL-17A, and IL-18) in the sample under test, the microcuvette-based chip was tested by filling the micro holes with a specific anti-interleukin antibody free in PBS solution (without any functionalization processes) at a fixed concentration of 7.3 fM and by dropping solutions at increasing concentrations of the interleukin (ranging from 5 aM to 50 fM), in a similar way to cortisol and estradiol detection in [23]. In particular, anti-IL-1β, anti-lL-18, and anti-IL-17A antibodies were employed to detect IL-1β protein, IL-18 protein, and IL-17A protein, respectively.

The resonance wavelength variations registered over time as Δλ_c_ arising from the binding events between the selected antibody and the respective interleukin were investigated using the measurement protocol described in Section 2.5.

**Figure 3:**
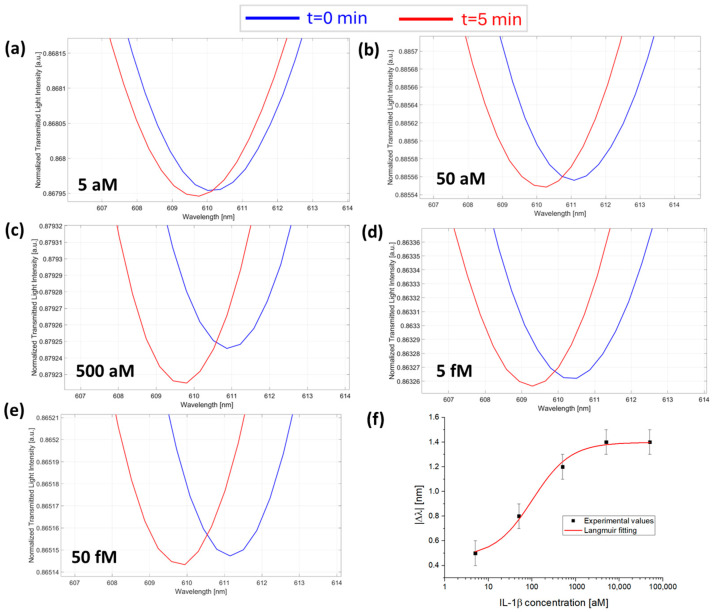
SPR spectra obtained at different IL-1β concentrations collected at zero (blue line) and five minutes (red line) of incubation time: (**a**) 5 aM; (**b**) 50 aM; (**c**) 500 aM; (**d**) 5 fM; and (**e**) 50 fM with the micro holes filled with the same solution at a fixed concentration of anti-IL-1β (7.3 fM) in PBS. (**f**) Absolute values of resonance wavelength variations (|Δλ|) at five minutes with respect to zero minutes (blank) as a function of the IL-1β concentration in a semi-log scale and Langmuir fitting of the experimental values. Mean values are out of n = 3, and error bars refer to the maximal measured standard deviation.

At first, the sensor response was monitored in the presence of a fixed concentration of anti-IL-1β for increasing concentrations of IL-1β in the sample. Figure 3a–e reports the SPR spectra monitored over time at different concentrations of IL-1β in the presence of 7.3 fM anti-IL-1β antibody (the micro holes were filled with a 1 µL of solution at a concentration value of 7.3 fM). As reported in Figure 3f, the absolute value of the resonance wavelength variations, calculated as Δλ_c_= λ_t5_ − λ_t0_ for each concentration, were plotted as a function of IL-1β concentrations and fitted with the Langmuir model equation reported in Equation (1). The Langmuir fitting parameters are listed in Table 1.

**Figure 4:**
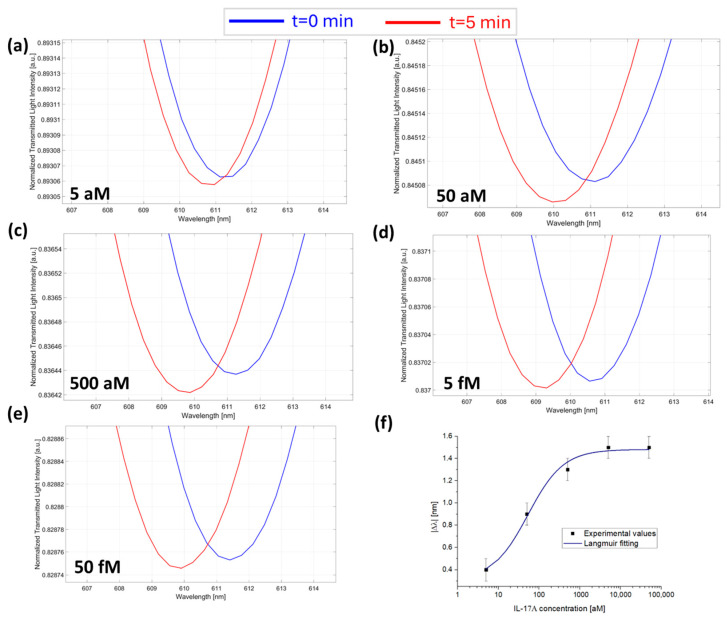
SPR spectra collected at zero (blue line) and five minutes (red line) of incubation time and obtained at different IL-17A concentrations: (**a**) 5 aM; (**b**) 50 aM; (**c**) 500 aM; (**d**) 5 fM; and (**e**) 50 fM with the micro holes filled with a solution at a fixed concentration of anti-IL-17A (7.3 fM) in PBS. (**f**) Absolute values of resonance wavelength variations (|Δλ|) at five minutes with respect to zero minutes as a function of the IL-17A concentration in a semi-log scale and Langmuir fitting of the experimental values. Mean values are out of n = 3, and error bars refer to the maximal measured standard deviation.

Then, the sensor response was tested for the anti-IL-17A antibody/IL-17A protein couple. Also in this case, the antibody concentration was maintained fixed at a value of 7.3 fM inside the micro holes, and IL-17A protein was tested in a concentration range from 5 aM to 50 fM by dropping the solution over the microcuvette chip (with the micro holes filled by anti-IL-17A antibody). Figure 4a–e reports the SPR spectra during time at different concentrations of IL-17A from 5 aM to 50 fM.

Also, in this case, the absolute value of Δλ_c_ (calculated as described above) for each concentration was plotted as a function of IL-17A concentrations and fitted with the Langmuir model equation (Equation (1)), as shown in Figure 4f. The fitting parameters of the Langmuir model are listed in Table 1.

**Figure 5:**
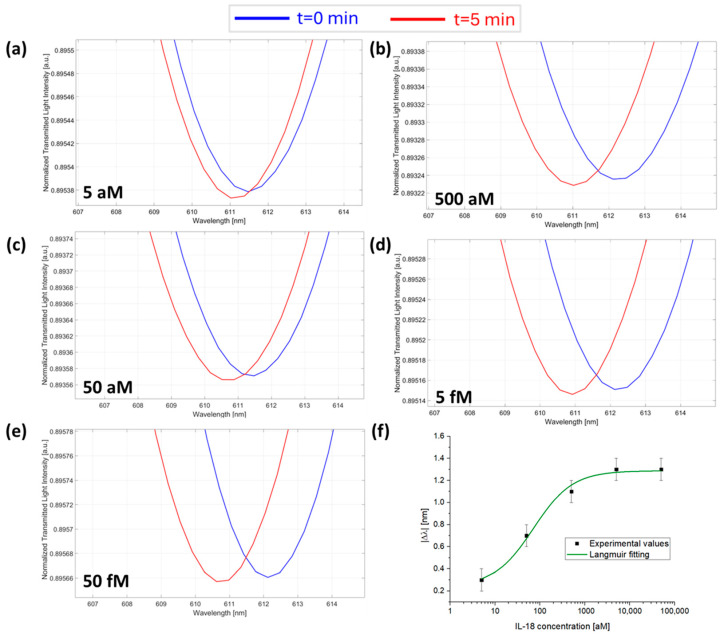
SPR spectra collected at zero (blue line) and five minutes (red line) of incubation time and obtained at different IL-18 concentrations: (**a**) 5 aM; (**b**) 50 aM; (**c**) 500 aM; (**d**) 5 fM; and (**e**) 50 fM with the micro holes filled with a solution at a fixed concentration of anti-IL-18 (7.3 fM) in PBS. (**f**) Absolute values of resonance wavelength variations (|Δλ|) at five minutes with respect to zero minutes as a function of the IL-18 concentration in a semi-log scale and Langmuir fitting of the experimental values. Mean values are out of n = 3, and error bars refer to the maximal measured standard deviation.

Finally, the sensor device response was investigated for the concentration monitoring of IL-18 protein, maintaining, as previously explained, a fixed concentration of anti-IL-18 antibody (7.3 fM) inside the micro holes of the microcuvette chip and dropping different concentrations of IL-18 ranging from 5 aM to 50 fM over the chip. Figure 5a–e reports the SPR spectra during time at different concentrations of IL-18. Also, in this test, |Δλ_c_| values registered over time and calculated as described above were plotted as a function of IL-18 concentrations and fitted with the Langmuir model equation (Equation (1)), as shown in Figure 5f, with the fitting parameters listed in Table 1.

In all the tested cases, as shown in Figure 3, Figure 4 and Figure 5, the resonance wavelength shifted toward lower values (blue shift) over the considered incubation time, in accordance with the results obtained in [23]. More specifically, the plasmonic wavelength shifts obtained with this device for all the antibody/interleukin couples considered in this work are ascribed to the modification of the propagated light caused by the phenomena taking place inside the micro holes, as extensively studied in previous works [23]. These could be ascribed to events attributable to the antibody/interleukin binding events, such as refractive index variations of the solution in the microcuvette, light absorption or emission phenomena from the receptor/analyte complex, and/or temperature variations due to heat released from the binding reaction, as demonstrated in [23]. All these events are proportionally linked to the number of binding events, which, over time, produce a modification of the modes of the propagated light, leading to changes in the resonance phenomena in the SPR-POF probe [23].

From the fitting parameters reported in Table 1, by using Equations (2)–(4), the POCT’s analytical parameters that characterize cytokine detection in PBS can be calculated as reported in Table 2 [26,27].

The obtained sensor parameters clearly show that the proposed microcuvette-based POCT shows high sensitivity, achieving LODs in the lowest attomolar range for all the cytokines tested. Similar performance in the attomolar range was previously reported in the case of IL-6 detection via a nanoplasmonic biochip [21]. However, the fabrication of nanoplasmonic chips requires high costs, high time consumption, and complex procedures (electron-beam lithography) performed by highly skilled personnel, in contrast to the fabrication of the microcuvette-based chip, which requires equipment and materials that have much lower costs. Moreover, a functionalization process is required in [21].

**Figure 6:**
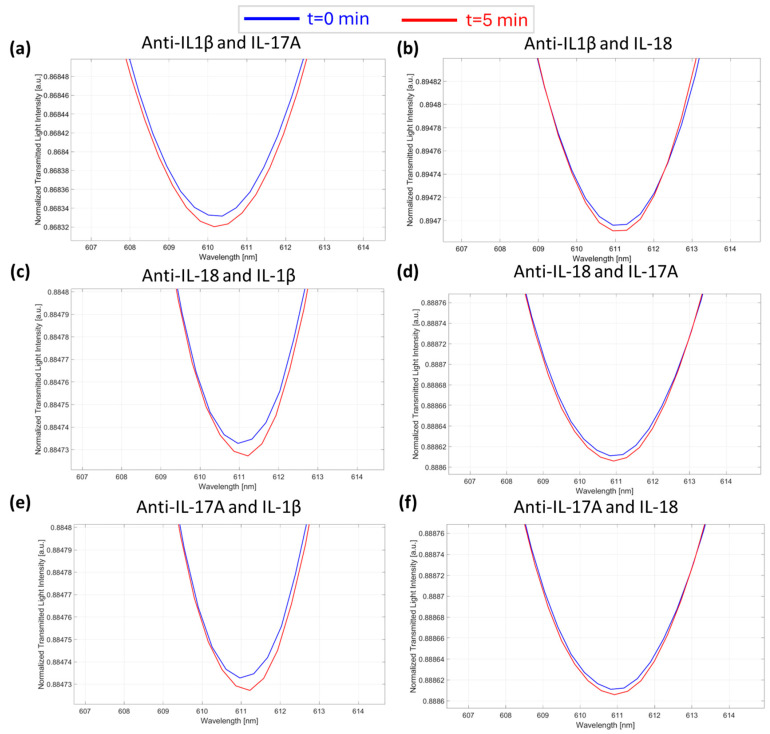
Selectivity tests. SPR spectra during time collected at zero (blue line) and five minutes (red line) of incubation time and obtained in the presence of the “wrong” anti-interleukin antibody–interleukin: (**a**) anti-IL-1β/IL-17A; (**b**) anti-IL-1β/IL-18; (**c**) anti-IL-18/IL-1β; (**d**) anti-IL-18/IL-17A; (**e**) anti-IL-17A/IL-1β; and (**f**) anti-IL-17A/IL-18.

This work also presents several tests to demonstrate the selectivity of the proposed device (POCT), which was evaluated by monitoring the POCT response in the presence of the “wrong” anti-interleukin antibody/interleukin couples. More specifically, the response of the microcuvette-based POCT was tested by filling the microcuvettes with a solution of anti-IL-1β antibody (specific for IL-1β) at a concentration of 7.3 fM and dropping onto the chip a solution of interleukins not specifically recognized by the antibody (i.e., IL-17A and IL-18) at a concentration of 1 pM (a high concentration value). The same procedure was repeated for all the antibodies considered (i.e., anti-IL-17A and anti-IL-18), which were tested under the same conditions in the presence of IL-1β and IL-18 for anti-IL-17A and in the presence of IL-1β and IL-17A for anti-IL-18. As shown in Figure 6, a negligible POCT response was achieved in all cases, despite the concentration of interfering cytokines being six orders of magnitude higher than those measured with the proposed device.

## 4. Conclusions

In this work, an innovative POCT based on unconventional plasmonic phenomena in POFs was tested for the detection of three cytokines in PBS at an attomolar level. The present work aims to demonstrate that the previously proposed sensing method [23], based on the interaction in a solution of intracellular bioreceptors and small molecules at low molecular weights, can be safely applied to different kinds of analyte–receptor interactions (e.g., interleukins), where the bioreceptor’s molecular weight (protein antibody) is roughly ten times that of the analyte (protein interleukin).

The POCT was made by a sensitive microcuvette chip produced by drilling three micro holes in a modified POF and an SPR-POF probe connected in series via a low-cost and simple setup. More specifically, the POCT was tested for three different cytokines, IL-1β, IL-17A, and IL-18, using three specific anti-interleukin antibodies. The antibody/interleukin interaction occurring free in PBS solution inside the micro holes avoided any kind of immobilization processes. In all the tested cases, the POCT response showed a LOD lower than 5 aM. The ultra-low concentration working range of the device allows the measurement of the expected load for highly diluted real samples, reducing the biofluid matrix effect. Therefore, the proposed POF-based POCT could be used as a novel class of laboratory instrumentation with unprecedented compactness, ultra-high sensitivity, and low-cost capabilities for several biomedical applications. With the advent of targeted interleukin therapies, the use of this technology may prove useful to identify patients who can benefit, monitor efficacy, optimize treatment strategies, and potentially identify non-responders [28,29,30].

## Figures and Tables

**Table 1:** Langmuir fitting parameters of cytokine detection in PBS.

Cytokines	|Δλ0|	|Δλmax|	K	Statistics	
	[nm]	[nm]	[aM]	Red. Χ2	Adj. R2
IL-1β	0.468 ± 0.02	1.397 ± 0.03	101.863 ± 24.2	0.144	0.991
IL-17A	0.306 ± 0.04	1.481 ± 0.04	53.244 ± 15.0	0.334	0.985
IL-18	0.241 ± 0.03	1.288 ± 0.03	71.013 ± 19.5	0.251	0.987

**Table 2:** Analytical parameters relative to cytokine detection in PBS via the proposed POCT.

Cytokines	LOD	S_lowc_	K_aff_
IL-1β	4.6 aM	0.014 nm/aM	0.01 (aM)^−1^
IL-17A	4.2 aM	0.028 nm/aM	0.02 (aM)^−1^
IL-18	4.8 aM	0.018 nm/aM	0.01 (aM)^−1^

## Data Availability

The data are available upon reasonable request from the corresponding author.
